# Supplementary material for: AffyMAPSDetector: a software tool to characterize Affymetrix GeneChip™ expression arrays with respect to SNPs
Source: BMC Bioinformatics. 2007 Jul 30;8:276. doi: 10.1186/1471-2105-8-276 (PMC1959249; doi:10.1186/1471-2105-8-276)
Supplement: Additional file 13 — AffyMAPSDetector v1 source code. AffyMAPSDetector source code. [file 1471-2105-8-276-S13.zip › AffyMAPSDetector_v1_src/doc/index-files/index-1.html]

A-Index


|  |  |  |  |
| --- | --- | --- | --- |
| **Overview** \| Package \| Class \| Use \| **Tree** \| **Deprecated** \| **Index** \| **Help** | | |  |
| PREV LETTER   **NEXT LETTER** | **FRAMES**    **NO FRAMES**     **All Classes** |  |  |


A B C D E F G H I J L M N O P R S T V W X Y 

---


## **A**

**ADDITIONAL\_GENE\_HEADERS** - Static variable in class affychip.snpdetector.model.SnpMatcher: **ADD\_TO\_FILTERED\_ROWS** - Static variable in class affychip.snpdetector.Constant: **ADD\_TO\_REMOVED\_ROWS** - Static variable in class affychip.snpdetector.Constant: **AffyMapsDetectorFrame** - class affychip.snpdetector.ui.AffyMapsDetectorFrame.: Title: Probe SNP Detector **AffyMapsDetectorFrame(JFrame)** - Constructor for class affychip.snpdetector.ui.AffyMapsDetectorFrame: It prepares the `DataState` of SNP Detector. **AffyMapsDetectorLauncher** - class affychip.snpdetector.AffyMapsDetectorLauncher.: Title: Probe SNP Detector **AffyMapsDetectorLauncher()** - Constructor for class affychip.snpdetector.AffyMapsDetectorLauncher: This is default contructor to create AffyMapsDetectorLauncher. **AffyMapsDetectorUI** - class affychip.snpdetector.ui.AffyMapsDetectorUI.: Title: Probe SNP Detector **AffyMapsDetectorUI()** - Constructor for class affychip.snpdetector.ui.AffyMapsDetectorUI: Default constructor. **AppIcons** - class affychip.snpdetector.resources.AppIcons.: Title: Probe SNP Detector **AppIcons()** - Constructor for class affychip.snpdetector.resources.AppIcons: **actionPerformed(ActionEvent)** - Method in class jwf.ErrorMessageBox: Handles the ok press. **actionPerformed(ActionEvent)** - Method in class jwf.Wizard: Handle's button presses. **addProbe(Probe)** - Method in class affychip.snpdetector.model.ProbeSet: **addSnpInfo(SnpInfo)** - Method in class affychip.snpdetector.model.GeneInfo: **addStatusEventListener(StatusEventListener)** - Method in class affychip.snpdetector.model.SnpMatcher: **addWizardListener(WizardListener)** - Method in class jwf.Wizard: Add a listener to this wizard. **affychip.snpdetector** - package affychip.snpdetector: **affychip.snpdetector.model** - package affychip.snpdetector.model: **affychip.snpdetector.model.event** - package affychip.snpdetector.model.event: **affychip.snpdetector.resources** - package affychip.snpdetector.resources: **affychip.snpdetector.ui** - package affychip.snpdetector.ui: **affychip.snpdetector.util** - package affychip.snpdetector.util

---


|  |  |  |  |
| --- | --- | --- | --- |
| **Overview** \| Package \| Class \| Use \| **Tree** \| **Deprecated** \| **Index** \| **Help** | | |  |
| PREV LETTER   **NEXT LETTER** | **FRAMES**    **NO FRAMES**     **All Classes** |  |  |


A B C D E F G H I J L M N O P R S T V W X Y 

---
